# Combination therapy of beta-blockers and digoxin is associated with increased risk of major adverse cardiovascular events and all-cause mortality in patients with atrial fibrillation: a report from the GLORIA–AF registry

**DOI:** 10.1007/s11739-024-03629-0

**Published:** 2024-05-23

**Authors:** Steven Ho Man Lam, Giulio Francesco Romiti, Brian Olshansky, Tze-Fan Chao, Menno V. Huisman, Gregory Yoke Hong Lip

**Affiliations:** 1 Liverpool Centre for Cardiovascular Science, University of Liverpool, Liverpool John Moores University and Liverpool Heart and Chest Hospital, Liverpool, UK; 2 Department of Translational Precision Medicine, Sapienza-University of Rome, Rome, Italy; 3 Division of Cardiology, Department of Medicine, University of Iowa, Iowa City, USA; 4 Division of Cardiology, Department of Medicine, Taipei Veterans General Hospital, Taipei, Taiwan; 5 Institute of Clinical Medicine, and Cardiovascular Research Center, National Yang Ming Chiao Tung University, Taipei, Taiwan; 6 Department of Thrombosis and Hemostasis, Leiden University Medical Center, Leiden, the Netherlands; 7 Danish Center for Health Services Research, Aalborg University, Aalborg, Denmark

**Keywords:** Atrial fibrillation, Mortality, Cardiovascular, Digoxin, Beta-blockers

## Abstract

The effect of digoxin and beta-blockers on cardiovascular outcomes and mortality remains unclear. The study aimed to determine differences in cardiovascular (CV) outcomes and death rates among patients with atrial fibrillation (AF) who were prescribed with beta-blockers, digoxin or combination therapy. Data from phase II/III of the prospective Global Registry on Long-Term Oral Anti-thrombotic Treatment in Patients with Atrial Fibrillation (GLORIA–AF) were analysed. The risk of major cardiovascular events (MACE) and death among patients with different prescriptions using COX proportional hazard regression was considered. Propensity score (PS) matching and weighting were further used to adjust for potential confounders of prescription use. A total of 14,201 patients [median age: 71.0 (IQR 64.0–77.0) years; 46.2% female] were recruited. After a median follow-up of 3.0 (IQR 2.4–3.1) years, 864 MACE, and 988 all-cause deaths were recorded. The incidence rate (IR) of MACE was 22.4 (95%CI 21.0–24.0) per 1000 person-years, while the IR of all-cause death was 25.4 (95%CI 23.8–27.0) per 1000 person-years. After multivariate adjustment with Cox regression, the risk of MACE (HR 1.35, 95% CI 1.09–1.68) and the risk of all-cause death (HR 1.28, 95%CI 1.04–1.57) were significantly higher in the combination therapy group, compared to the beta-blockers alone group. The risks of MACE and all-cause death remained significant in both PS matched and PS weighted cohort Among AF patients, combination therapy of beta-blockers and digoxin was associated with higher risks of MACE and all-cause death compared to beta-blockers alone.

**Supplementary Information:**

The online version contains supplementary material available at 10.1007/s11739-024-03629-0.

## Introduction

Safe and effective atrial fibrillation (AF) management is crucial to attenuate cardiovascular burden among patients with AF. Controlling heart rate with antiarrhythmic drugs is one of the measures in AF management and is recommended in guidelines for most patients with AF to alleviate symptoms and reduce CV events [1]. Both beta-blockers and digoxin can be effective for controlling heart rate [2]. Numerous trials and studies have proved the efficacy of beta-blockers for controlling heart rate in patients with heart failure and/or AF [3–5]. While beta-blockers are also effective in improving clinical outcomes, including all-cause mortality and major adverse cardiovascular events among patients with heart failure (HF), the effectiveness among patients with AF was not established [6–8]. An individual patient meta-analysis of clinical trials reported that beta-blockers reduced mortality in patients with HF in sinus rhythm, but not in HF patients with AF [9].

While digoxin is generally viewed as a second line therapy in patients with unsatisfactory rate control using beta-blockers, its role among patients with AF remains controversial [1]. Meta-analyses of clinical trials and observational studies both raised concern about digoxin’s unfavourable impact on clinical outcomes including mortality among patients with AF [10, 11]. On the other hand, patients with heart failure receiving a combination of a beta-blocker and an angiotensin-converting enzyme inhibitor/angiotensin receptor blocker had lower risks of cardiovascular death, compared to patients receiving only one drug [12]. However, the effect of digoxin as opposed to beta-blockers and the effect of combination therapy on cardiovascular outcome and mortality in patients with AF have not yet been fully elucidated.

We hypothesised that cardiovascular outcomes and death rates would differ among patients with AF based on beta-blockers and digoxin prescriptions. The present analysis using a large, prospective global registry of newly diagnosed AF patients aimed to reveal differences in the cardiovascular outcomes and mortality among different prescriptions of beta-blockers, digoxin and combination therapy in patients with AF.

## Methods

### Study design

We performed an analysis of the cohort data from the Global Registry on Long-Term Oral Antithrombotic Treatment in Patients with Atrial Fibrillation (GLORIA–AF) database. Full details on the protocol and methodology of GLORIA–AF had been published previously [13]. In brief, GLORIA–AF is a global, multicentred prospective registry programme made up of three phases which sought to evaluate the long-term safety and effectiveness of AF therapy among recently diagnosed AF patients. Patients aged 18 years or older with new-onset non-valvular AF and CHA_2_DS_2_–VASc score ≥ 1 were recruited in GLORIA–AF. Detailed inclusion and exclusion criteria were described elsewhere. Phase I was conducted before the approval of dabigatran with no data collected beyond the baseline visit. In phase II, patients were followed for 2 years if they received dabigatran, while in phase III, patients were followed for 3 years irrespective of their antithrombotic treatment. Ethics approvals were obtained from local institutional review boards and written informed consents were obtained from patients. The study was carried out in accordance with the Declaration of Helsinki.

### Data collection

Patients who were prescribed beta-blockers and/or digoxin from Phase II and Phase III of GLORIA–AF were included in the study population. Standardised, specifically designed data collection tools were used to collect data on demographics, comorbidities and therapies at enrolment across all sites around the globe. AF was classified according to the European Society of Cardiology recommendations and severity of AF-related symptoms was determined using the European Heart Rhythm Association classification. [14]

### Study outcomes

The primary outcome of interest was major adverse cardiovascular events (MACE) defined as the composite of cardiovascular death, stroke, and myocardial infarction. The secondary outcome was all-cause mortality. All outcomes were diagnosed and input by physicians.

### Statistical analysis

Continuous variables were presented as median and Interquartile range (IQR), while categorical variables were presented as count and percentage. Differences in the baseline characteristics between groups were analysed with Mann–Whitney *U* test, and Chi-squared test for continuous variables and categorical variables, respectively. The incidence rates (IRs) per 1000 patient-years of MACE and all-cause mortality were statistically inferred from Poisson distribution and were compared among different groups of drug users using Poisson regression. The number needed to treat for harm (NNT_harm_) were calculated by the inverse of the absolute risk increase in the treatment group (beta-blockers and/or digoxin), compared to the control group (beta-blockers only) and the treatment group. The association between the outcomes and the explanatory variables were analysed by Cox proportional hazard regression. The results were expressed as hazard ratio (HR) and 95% confidence interval (CI). The selection of explanatory variables was based on previous evidence and clinical judgement. Kaplan–Meier survival curves and log-rank test were also produced to illustrate the differences in the survival distributions among different groups of digoxin and beta-blockers users and to determine the significance of the differences, respectively.

#### Propensity score matching and weighting

Propensity score (PS) matching and PS weighting were performed to further control confounders which may affect the prescription choice. PS was used to recreate a cohort in which the baseline confounders were balanced among patients receiving beta-blockers/digoxin/beta-blockers & digoxin. The PS for receiving beta-blockers or digoxin or the combination of beta-blockers and digoxin were computed using a generalised linear model with logistic regression in which the prescriptions were included as the dependent variables and other variables are included as covariates. 1:1 nearest neighbour matching was used to generate the matched cohort based on the calculated PS. Weighted cohort was generated by inverse probability weighting (IPW) which was the process of creating pseudo-population in which the exposure (i.e., different drugs) is independent of the measured confounders. IPW uses the propensity score to balance the characteristics across exposure groups with weight given to each sample in the analysis according to the inverse probability of receiving the actual exposure. In our analysis, stabilised weight was calculated and assigned to each participant according to the participant’s probability of receiving his/her own treatment regimen (Beta-blockers alone vs Digoxin alone vs Beta-blockers & Digoxin) which was created by generalised linear model. The formulae of the stabilised weights for the exposed and unexposed group could be found in the supplementary data [15].

The adjusted exposure to the different treatment regimen was modelled into the Cox regression. We also assessed the robustness of covariate balance by measuring standardised mean differences of each covariate across different exposure groups, expressed as a percentage of the pooled standard deviation. Love plots were generated to illustrate the standardised differences before and after PS matching or PS weighting. A covariate with a standardised difference less than 0.1 across the exposure group was generally considered to be well-balanced [16]. All statistical analyses were computed with R version 4.3.1.

## Results

### Baseline characteristics

A total of 14,201 patients [median age: 71.0 (IQR 64.0–77.0) years; 46.2% female] who were prescribed with beta-blockers and/or digoxin were included in the study. Supplementary Fig. 1 shows how the study cohort was assembled from the GLORIA–AF database. Table 1 shows the baseline characteristics of the study cohort and compared the characteristics of the different groups of beta-blockers and/or digoxin prescription.

**Table 1:** Baseline characteristics and the comparison among different groups of beta-blockers and/or digoxin users

	Whole cohort *n* = 14,201	Beta-blockers only *n* = 12,455	Digoxin only *n* = 531	Both Beta-blockers and Digoxin *n* = 1215	*p* value
Age, years, median (IQR)	71.0 (64.0–77.0)	71.0 (64.0–77.0)	74.0 (67.0–80.0)	70.0 (62.0–78.0)	< 0.001*
Sex, female, *n* (%)	6554 (46.2)	5712 (45.9)	278 (52.4)	564 (46.4)	0.013*
BMI, median (IQR)	28.0 (24.9–32.1)	28.0 (24.9–32.1)	27.0 (23.9–31.2)	28.2 (24.9–32.5)	< 0.001*
Hypertension, *n* (%)	10,999 (77.5)	9788 (78.6)	339 (63.8)	872 (71.8)	< 0.001*
Diabetes mellitus, *n* (%)	3379 (23.8)	3016 (24.2)	95 (17.9)	268 (22.1)	0.001*
Hyperlipidaemia, *n* (%)	6140 (43.2)	5547 (44.5)	152 (28.6)	441 (36.3)	< 0.001*
Smoking status, *n* (%)					0.580
Never smoked	8313 (58.5)	7319 (58.7)	309 (58.2)	685 (56.4)	
Ex-smoker	4560 (32.1)	3979 (31.9)	174 (32.8)	407 (33.5)	
Current smoker	1328 (9.4)	1156 (9.3)	48 (9.0)	123 (10.1)	
Alcohol drinking status, *n* (%)					< 0.001*
No alcohol	6349 (44.7)	3368 (27.0)	104 (19.6)	334 (27.5)	
< 1 drink/week	3806 (26.8)	5542 (44.5)	291 (54.8)	516 (42.5)	
1–7 drinks/week	3023 (21.3)	2684 (21.5)	90 (16.9)	249 (20.5)	
≥ 8 drinks/week	1023 (7.2)	861 (6.9)	46 (8.7)	116 (9.5)	
CHF, *n* (%)	3663 (25.8)	2789 (22.4)	223 (42.0)	651 (53.6)	< 0.001*
CAD, *n* (%)	3061 (21.6)	2733 (21.9)	75 (14.1)	253 (20.8)	< 0.001*
Stroke	1415 (10.0)	1264 (10.1)	55 (10.4)	96 (7.9)	0.042*
AKF, *n* (%)	252 (1.8)	219 (1.8)	14 (2.6)	19 (1.6)	0.274
COPD, *n* (%)	854 (6.0)	679 (5.5)	65 (12.2)	110 (9.1)	< 0.001*
Pacemaker insertion, *n* (%)	707 (5.0)	636 (5.1)	12 (2.3)	59 (4.9)	0.012*
OAC, *n* (%)	12,416 (87.4)	10,850 (87.1)	460 (86.6)	1106 (91.0)	< 0.001*
Antiplatelet, *n* (%)	3651 (25.7)	3306 (26.5)	95 (17.9)	250 (20.6)	< 0.001*
ACE-inhibitors, *n* (%)	4761 (33.5)	4123 (33.1)	133 (25.0)	505 (41.6)	< 0.001*
Diuretic, *n* (%)	6089 (42.8)	4978 (40.0)	295 (55.6)	816 (67.2)	< 0.001*
Heart Rate, median (IQR)	76.0 (65.0–90.0)	75.0 (65.0–89.0)	82.0 (72.0–96.00	84 (71.0–101.0)	< 0.001*
AF classification, *n* (%)					< 0.001*
-Paroxysmal	7635 (53.8)	7066 (56.7)	161 (30.3)	408 (33.6)	
-Persistent	5092 (35.9)	4251 (34.1)	238 (44.8)	603 (49.6)	
-Permanent	1474 (10.4)	1138 (9.1)	132 (24.9)	204 (16.8)	
Digoxin and Beta-blockers status, *n* (%)		N/A	N/A	N/A	N/A
Beta-blockers only	12,455 (87.7)				
Digoxin only	531 (3.7)				
Both Beta-blockers and Digoxin	1215 (8.6)				

*BMI* Body Mass Index, *CHF* Congestive Heart Failure, *CAD* Coronary Artery Disease, *AKF* Abnormal Kidney Function, *COPD* Chronic Obstructive Pulmonary Disease, *OAC* Oral Anti-Coagulant, *ACE* angiotensin-converting-enzyme, *AF* atrial fibrillation, *N/A* not applicable

History of congestive heart failure (53.6% vs 22.4%), Heart rate (median: 84 vs 75) and use of diuretics (67.2% vs 40.0%) were higher in the group with combination therapy, compared to the group with beta-blockers prescription alone. After PS matching, all baseline covariates were well-balanced between beta-blockers and combination therapy with a standardised mean difference less than 0.1 as shown in the Love plot (Supplementary Fig. 2). In the PS weighted cohort, a similar balance was also achieved among the baseline covariates (Supplementary Fig. 3).

### Incidence of MACE and all-cause mortality

After a median follow-up period of 3.0 (IQR 2.4–3.1) years, 864 MACEs and 988 all-cause deaths were recorded. Among 14,201 patients, 864 MACE events occurred during 38,491 person-years of follow-up which corresponded to an IR of 22.4 (95%CI 21.0–24.0) per 1000 person-years, while the IR of all-cause death was 25.4 (95%CI 23.8–27.0) per 1000 person-years.

In the original cohort, 723 (5.8%), 35 (6.6%) and 106 (8.7%) cases of MACE developed among the group with beta-blocker prescription alone, digoxin prescription alone and combination therapy, respectively. The incidence of MACE was significantly higher (*p* < 0.001) in the combination therapy group, compared to the beta-blockers only group (Table 2). All-cause mortality was 812 (6.5%), 53 (10.0%), 123 (10.1%) in the beta-blocker group, digoxin group, and combination therapy group, respectively. The incidence of all-cause mortality was significantly higher in both the digoxin group and the combination therapy group, compared to the beta-blockers only group (Table 2).

**Table 2:** Incidence rate and incidence rate ratio among different treatment groups

	IR of MACE per 1000 person-years (95% CI)	IR of death per 1000 person-years (95% CI)	IRR of MACE	*p* value	IRR of death	*p* value
Beta-blockers	21.3 (19.8–22.9)	23.6 (22.0–25.3)	Ref	N/A	Ref	N/A
Digoxin	25.4 (17.7–35.4)	38.3 (28.7–50.1)	1.20 (0.84–1.66)	0.303	1.63 (1.22–2.12	0.001*
Beta-Blockers plus Digoxin	33.6 (27.5–40.7)	38.5 (32.0–45.9)	1.58 (1.28–1.93)	< 0.001*	1.63 (1.34–1.96)	< 0.001*

*IR* incidence rate, *IRR* incidence rate ratio, *N/A* not applicable

### NNT_harm_ with digoxin and beta-blockers

In the original cohort, NNT_harm_ to get one additional MACE in the digoxin alone group and the combination therapy group was 125 (95%CI: reached infinity and became not significant) and 35 (95% CI 21.4–71.5), respectively. NNT_harm_ to get one additional all-cause death was 29 (95% CI 15.8–87.2) in the digoxin alone group and 28 (95% CI 18.3–50.8) in the combination therapy group.

In the PS matched cohort, NNT_harm_ to get one additional MACE in the digoxin alone group and the combination therapy group was 266 (95% CI: reached infinity and became not significant) and 45 (95% CI 22.3–878.8), respectively. NNT_harm_ to get one additional all-cause death was 255 (95% CI not significant) in the digoxin alone group and 38 (95% CI 20.4–261.5) in the combination therapy group.

### The risk of MACE with digoxin and beta-blockers

**Fig. 1:**
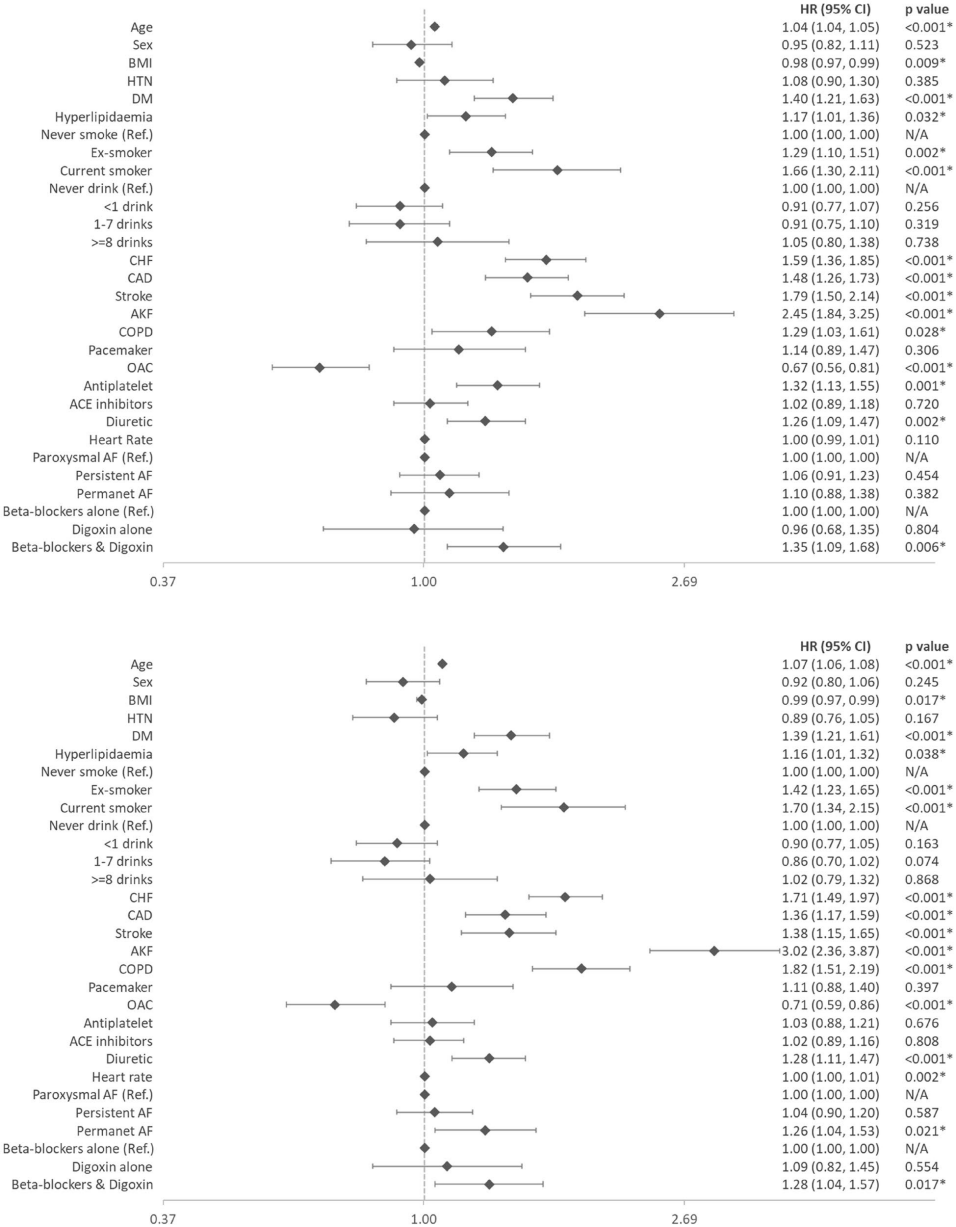
Forest plot illustrating the multivariate Cox regression for predicting MACE (top) and all-cause mortality (bottom) in the whole cohort. *BMI* Body Mass Index, *HTN* Hypertension, *DM* Diabetes Mellitus, *CHF* Congestive Heart Failure, *CAD* Coronary Artery Disease, *AKF* Abnormal Kidney Function, *COPD* Chronic Obstructive Pulmonary Disease, *OAC* Oral Anti-Coagulant, *ACE* Angiotensin-converting-enzyme, *AF* Atrial fibrillation

After multivariate adjustment with Cox regression, the risk of MACE (HR: 1.35, 95% CI 1.09–1.68) was significantly higher in the combination therapy group, compared to the beta-blockers only group. However, the risks were not significantly different between the beta-blocker only group and the digoxin only group in the original cohort (Fig. 1).

**Fig. 2:**
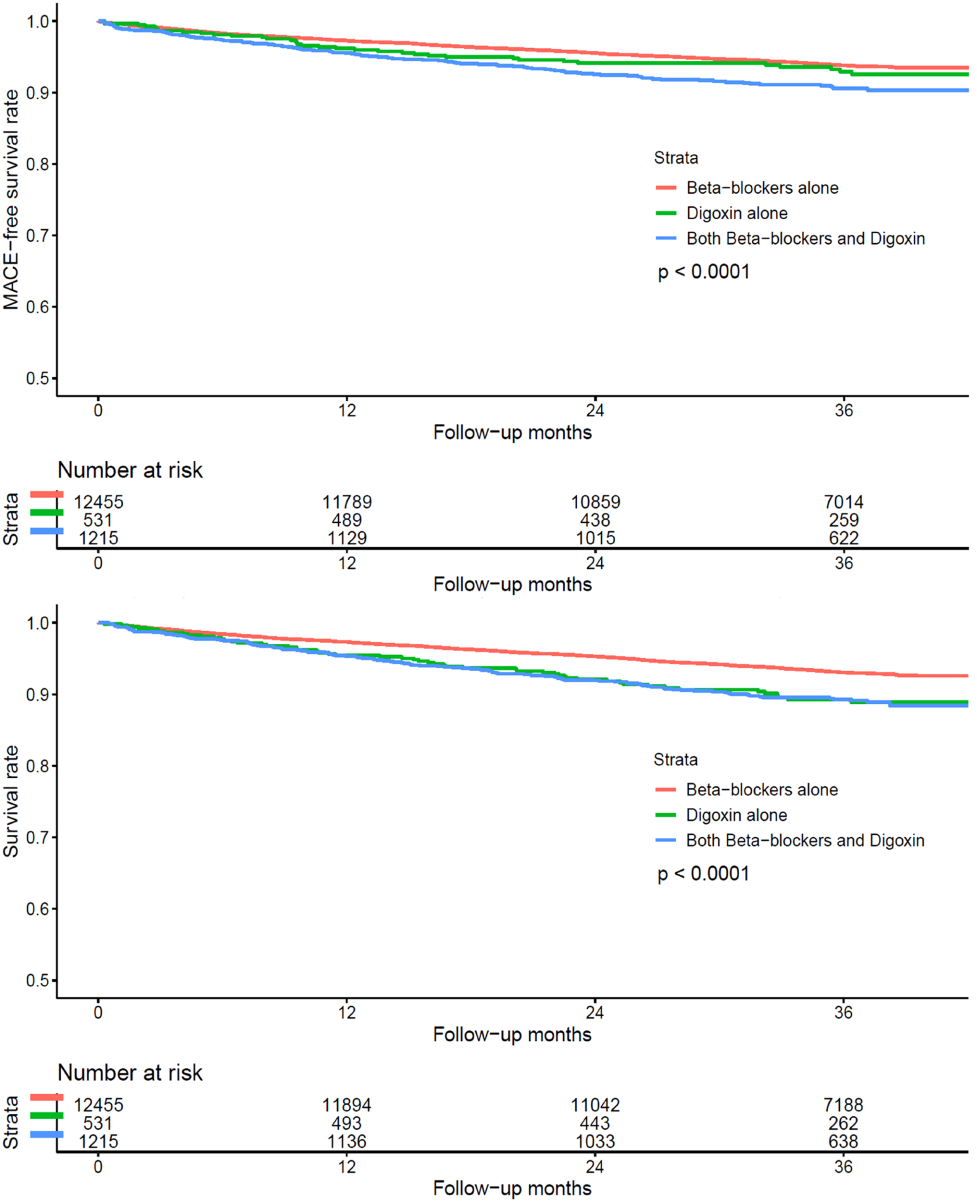
Kaplan-Meier Survival Curves for illustrating the MACE-free survival distribution (top) and the survival distribution (bottom) among different groups of drug users

The elevated risk in the combination therapy remained significant in both the PS matched cohort (HR 1.43, 95% CI 1.06–1.92) and the PS weighted cohort (HR 1.57, 95% CI 1.17–2.11) (Table 3). The survival distribution significantly differed among different drug groups as shown by the Kaplan–Meier survival curves and the log-rank test (*p* < 0.001) with the combination therapy group having the lowest MACE-free survival rate (Fig. 2).

**Table 3:** Multivariate Cox regression for predicting risk of MACE and all-cause mortality in the propensity score matched and propensity score weighted cohort

	Propensity score matched cohort^				Propensity score weighted cohort^a^			
	MACE HR (95% CI)	*p* value	All-cause mortality HR (95% CI)	*p* value	MACE HR (95% CI)	*p* value	All-cause mortality HR (95% CI)	*p* value
BB alone	Ref		Ref		Ref		Ref	
DG alone	0.98 (0.61–1.57)	0.936	1.13 (0.77–1.68)	0.529	0.86 (0.53–1.40)	0.537	1.24 (0.77–2.00)	0.379
BB & DG	1.43 (1.06–1.92)	0.017*	1.42 (1.08–1.86)	0.013*	1.57 (1.17–2.11)	0.003*	1.33 (1.01–1.75)	0.043*

*BB* beta-blockers, *DG* digoxin

^a^Adjusted for the same variables in the original cohort

### The risk of all-cause death with digoxin and beta-blockers

In the original cohort, the risk of all-cause death (HR 1.28, 95% CI 1.04–1.57) was significantly higher in the combination therapy group, compared to beta-blockers only group (Fig. 1). The observed increase in the risk of all-cause death remained significant in the PS matched cohort (HR 1.42, 95% CI 1.08–1.86) (Table 3). The risk of all-cause death in the combination therapy group (HR 1.33, 95% CI 1.01–1.75) were still significantly higher, compared to the beta-blockers only group in the PS weighted cohort. However, the risks were not significantly different between the beta-blockers only group and the digoxin only group after multivariate adjustment.

Kaplan–Meier survival curves and log-rank test demonstrated the significant differences in the survival rates among different groups of drug prescription (*p* < 0.001). The survival rate was the lowest in the combination therapy group (Fig. 2).

### Post-hoc interaction analysis

To further investigate if the increased risk of death associated with digoxin and/or beta-blockers was influenced by HF, abnormal kidney function, and angiotensin-converting enzyme inhibitors (ACEIs) or angiotensin II receptor blockers (ARBs), a post-hoc interaction analysis was performed to evaluate if the risk was modified by different sub-groups of patients. Supplementary Fig. 4 summarises the analysis and the result indicated that the risk was not different between patients with or without HF, patients with or without abnormal kidney function, and patients with or without ACEIs or ARBs (*p* > 0.05).

## Discussion

In this analysis from a large, prospective global cohort of AF patients, our principal finding was that over a median of 3 year follow-up, significantly higher risks of MACE and all-cause mortality were found in the combination therapy group, compared to the group with beta-blockers alone in both the original cohort and the PS matched/weighted cohort. Our observation suggests caution when using beta-blockers and digoxin as combination therapy, for treating patients with AF.

The safety concern of digoxin has been raised and discussed before. The evidence for safety of digoxin has been mixed. Some studies suggested an increased risk of mortality related to digoxin use, and in particular, a dose–response relationship related to the serum digoxin concentration in patients with HF or AF [17, 18]. While some meta-analyses revealed a neutral association between digoxin and mortality in patients with HF or AF [10, 19], others have suggested an increased risk associated with digoxin use [11]. One recent umbrella review of systematic reviews and meta-analyses concluded that digoxin is harmful to the survival of patients with HF or AF [20].

In this study, 14.0% of patients were prescribed with digoxin alone or in combination with beta-blockers. It may reflect the fact that sicker patients with higher heart rate at baseline were given digoxin together with beta-blockers for effective control of heart rate. Though the risks of MACE and all-cause mortality in the group with digoxin alone did not increase, when digoxin was given in combination with beta-blockers, the risks of MACE and all-cause mortality increased when compared to beta-blockers alone. In addition, the results from the PS matched and weighted cohort suggested that after neutralising the known exposure biases to different treatment regimens, the risks of MACE and all-cause mortality remained higher in the group with combination therapy compared to the group with beta-blockers only. The post-hoc interaction analysis also indicated that the increased risk of death associated with digoxin and/or beta-blockers was not modified by HF, abnormal kidney function, and ACEIs/ARBS.

The result suggested that the prescription of beta-blockers together with digoxin maybe harmful to the cardiovascular health and survival among patients with AF. Of note, there have been limited data about the risk or benefit of digoxin prescription on top of beta-blockers among participants with AF with respect to cardiovascular and mortality outcomes. In a recently published study, the risks of cardiovascular hospitalisation and mortality were not increased in patients treated with either digoxin or combination of digoxin and beta-blockers therapy compared to patients treated with only beta-blockers [21]. However, the study had a short follow-up duration with a median of 356 days compared to a median of 3 years in our study. In addition, only PS weighting was performed in their study, while we have also performed PS matching and the results from the PS weighted and the PS matched cohorts were consistent.

In general, our results were compatible with previous studies which had suggested a harmful effect of digoxin among patients with AF. Some prior studies have demonstrated the association between digoxin and all-cause death in patients with HF and/or AF [17, 18, 22, 23]. In particular, an ancillary analysis from the ARISTOTLE trial highlighted the dose–response relationship between serum digoxin concentration and the risk of death among AF patients, whereby for every 0.1 ng/ml increase in serum digoxin concentration, a 4% higher risk of overall mortality (HR 1.04, 95% CI 1.01–1.06) was seen [18]. Although, there were no significant association between digoxin and stroke/myocardial infarction, there was a significant association between serum digoxin concentration and cardiovascular death (HR 1.24, 95% CI 1.08–1.42) for every 0.5 ng/ml increase in digoxin concentration. In addition, in the PALLAS trial, serum digoxin concentration was significantly higher in AF patients taking dronedarone which suggested the possibility of drug interaction [24].

In the post-hoc analysis of the ROCKET AF study, digoxin was associated with a significant increase in all-cause mortality (HR 1.17, 95% CI 1.04–1.32), vascular death (HR 1.9, 95% CI 1.03–1.39) in patients with AF after adjusting for baseline characteristics [25]. Data from a Health Insurance database also showed that the risk of mortality (HR 1.12, 95% CI 1.10–1.14) and the risk of ischemic stroke (HR: 1.41, 95%CI 1.01–1.44) significantly increased in AF patients who received digoxin, compared to patients who did not received any rate-control drug [26, 27]. In a more recent cohort with a smaller sample size (*n* = 1376), AF patients treated with digoxin experienced significantly higher risks of all-cause (HR 1.39, 95% CI 1.11–1.73), cardiovascular (HR 1.44, 95% CI 1.13–1.82) and arrhythmic (HR 2.03, 95% CI 1.63–2.54) death when compared to AF patients not treated with digoxin [22]. A recent umbrella review of 12 systematic reviews and/or meta-analyses also concluded that digoxin may increase the risk of all-cause and cardiovascular death in patients with AF [20]. Note that an umbrella review is on the top of the hierarchy of evidence synthesis methods [28]. In the review, over 4,500,000 patients from 12 meta-analyses of observational studies were included, and suggested digoxin was associated with an elevated risk of all-cause mortality (HR 1.19, 95% CI 1.14–1.25) and cardiovascular mortality (HR 1.19, 95% CI 1.06–1.33) with a moderate certainty of evidence [20].

In the atrial fibrillation better care (ABC) holistic pathway, better symptom control is one of the pillars in the management guideline of AF. The latest guideline recommends the prescription of both beta-blockers and digoxin in patients with HF and reduced ejection fraction (HFrEF) but does not prefer digoxin over non-dihydropyridine calcium channel blockers as the second-line treatment in patients with HF and preserved ejection fraction (HFpEF) [1]. The result suggests caution when using digoxin in addition to beta-blockers and highlight the risk that need to be considered when choosing the second-line drug for better rate control in patients with HFpEF.

## Limitations

Our study does have limitations. First, despite extensive adjustment for potential confounders using PS matching and weighting, the analysis did not include certain important confounders e.g. left ventricular ejection fraction, severity of underlying diseases and the extent of coronary artery disease. There were residual factors which may confound the result and hence the result should be interpreted with caution. Second, only prescription at baseline was captured, but not the dosages and the patients’ adherence to the prescription. In addition, subsequent prescriptions of the drugs during follow-up were not included. Third, no specific digoxin/beta blockers dosing guidelines were provided by the GLORIA–AF protocol. We also lacked the laboratory data on the individuals’ serum digoxin concentrations. Hence, the dosages were likely to be varied among participants and the dose-dependent effect could not be investigated in our analysis.

## Conclusion

Among patients with AF, the risks of MACE and all-cause death were significantly higher in patients with combined prescription of beta-blockers and digoxin, compared to patients with a single prescription of beta-blockers. Our results underscore the potential safety issues of the use of digoxin and a review of digoxin’s role among AF patients.

### Supplementary Information

Below is the link to the electronic supplementary material.

Supplementary file1 (DOCX 1118 KB)

## Data Availability

All data generated or analysed during this study are included in this published article and its supplementary information files.
