# Supplementary material for: Combination therapy of beta-blockers and digoxin is associated with increased risk of major adverse cardiovascular events and all-cause mortality in patients with atrial fibrillation: a report from the GLORIA–AF registry
Source: Intern Emerg Med. 2024 May 23;19(5):1369–78. doi: 10.1007/s11739-024-03629-0 (PMC11364656; doi:10.1007/s11739-024-03629-0)
Supplement: Supplementary file 1 — Supplementary file1 (DOCX 1118 KB) [file 11739_2024_3629_MOESM1_ESM.docx]

**Supplementary Figures**


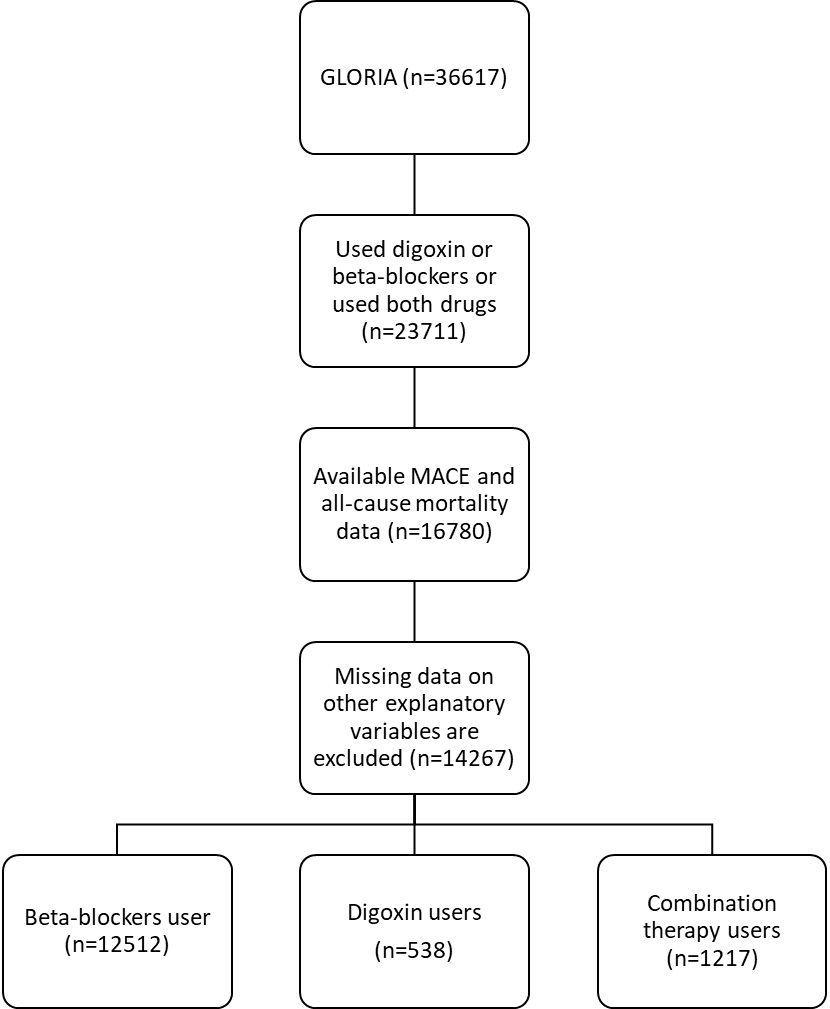


**Supplementary figure 1. Flow chart showing the assembly of the study cohort.**


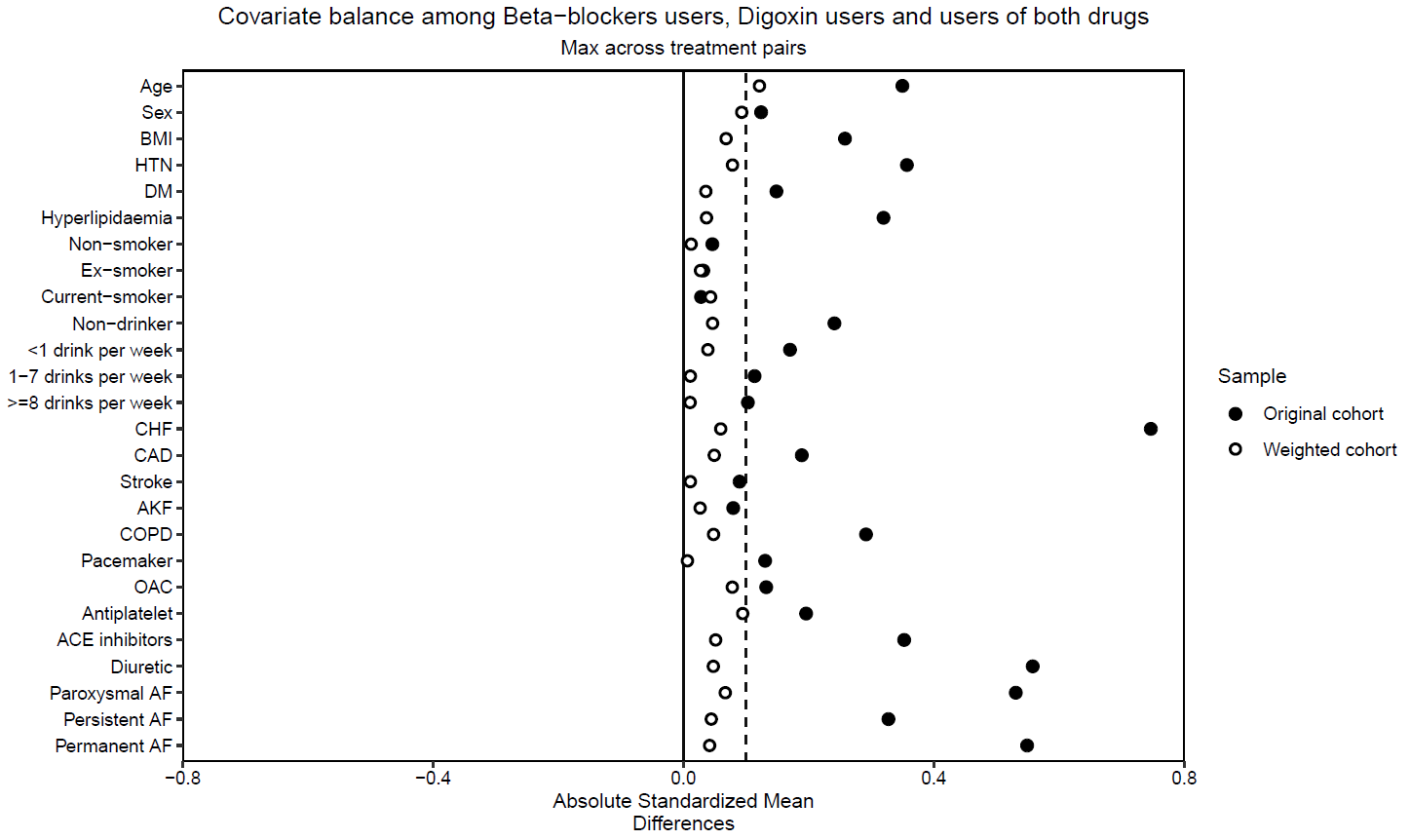


**Supplementary figure 3. Covariate balance after propensity score weighting among beta-blockers users, digoxin users and users of both beta-blockers and digoxin.** BMI, Body Mass Index; HTN, Hypertension; DM, Diabetes Mellitus; CHF, Congestive Heart Failure; CAD, Coronary Artery Disease; AKF, Abnormal Kidney Function; COPD, Chronic Obstructive Pulmonary Disease; OAC, Oral Anti-Coagulant; AF, Atrial fibrillation

**Supplementary data**

$${Weight}_{exposed group}= \frac{Proportion exposed}{Propesnsity score}$$

$${Weight}_{unexposed group}= \frac{Proportion unexposed}{\left( 1-Propesnsity score \right)}$$

Supplementary formula for calculating the stabilised weight.


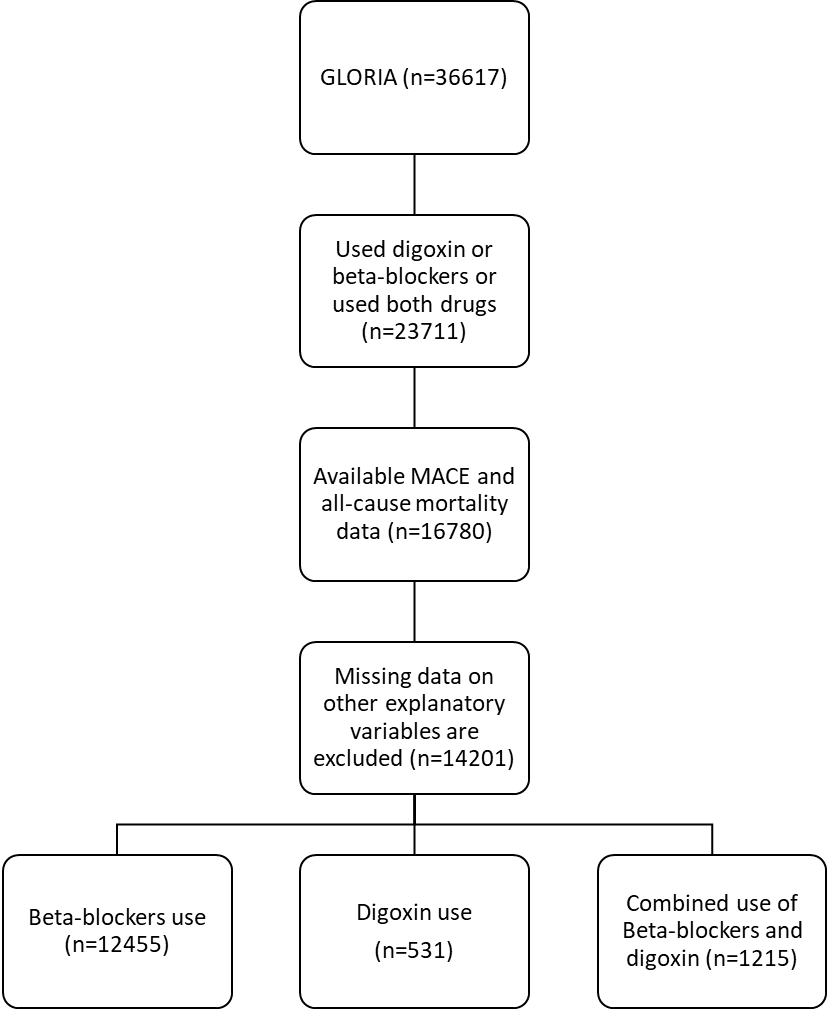


Supplementary figure 1. Flow chart showing the assembly of the study cohort


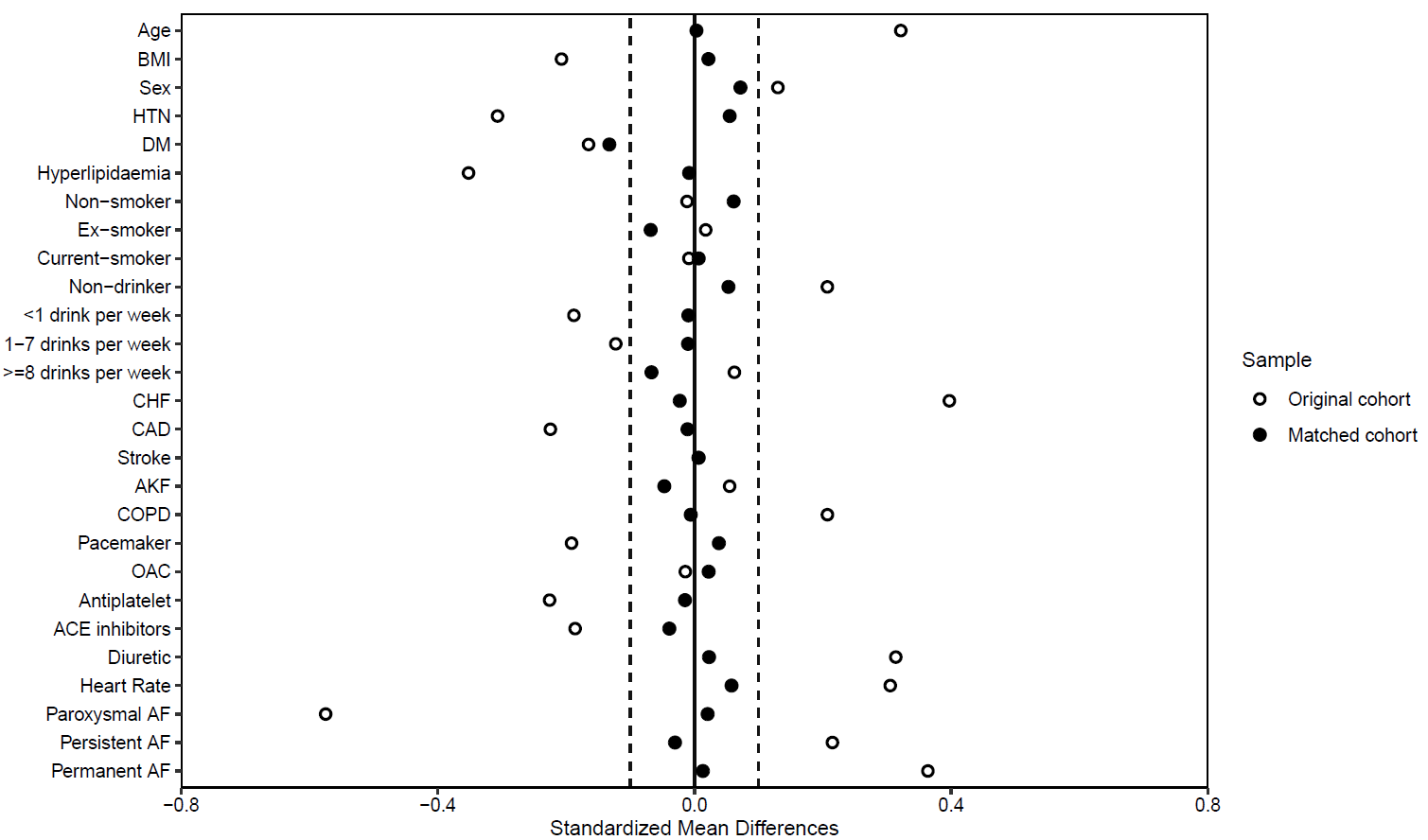

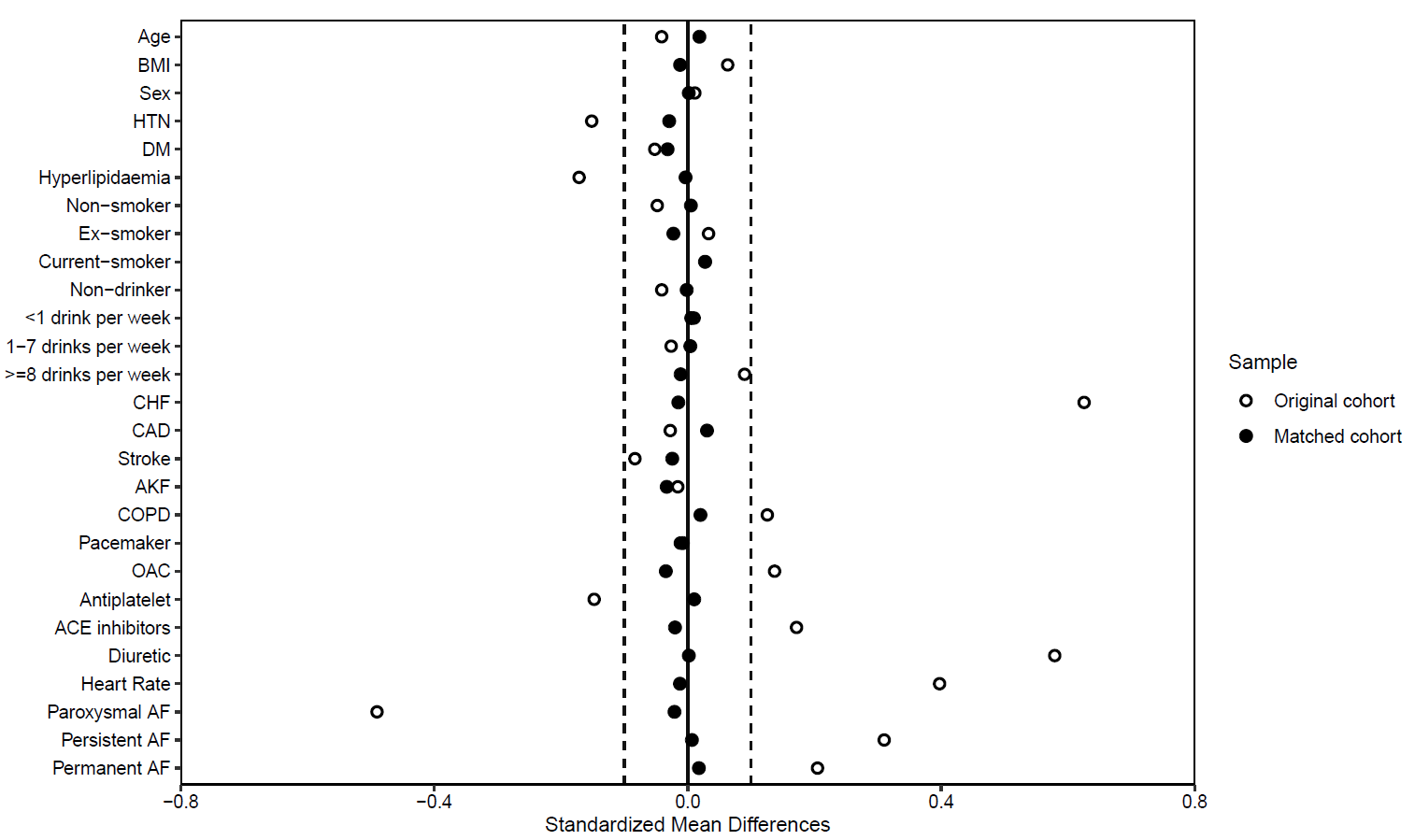


**Supplementary figure 2. Covariate balance between beta-blockers users and digoxin users (top), and between beta-blockers users and users of both beta-blockers and digoxin (bottom).** BMI, Body Mass Index; HTN, Hypertension; DM, Diabetes Mellitus; CHF, Congestive Heart Failure; CAD, Coronary Artery Disease; AKF, Abnormal Kidney Function; COPD, Chronic Obstructive Pulmonary Disease; OAC, Oral Anti-Coagulant; ACE, Angiotensin-converting-enzyme; AF, Atrial fibrillation

**Supplementary figure 3. Covariate balance among beta-blockers users, digoxin users and users of both beta-blockers and digoxin.** BMI, Body Mass Index; HTN, Hypertension; DM, Diabetes Mellitus; CHF, Congestive Heart Failure; CAD, Coronary Artery Disease; AKF, Abnormal Kidney Function; COPD, Chronic Obstructive Pulmonary Disease; OAC, Oral Anti-Coagulant; ACE, Angiotensin-converting-enzyme; AF, Atrial fibrillation


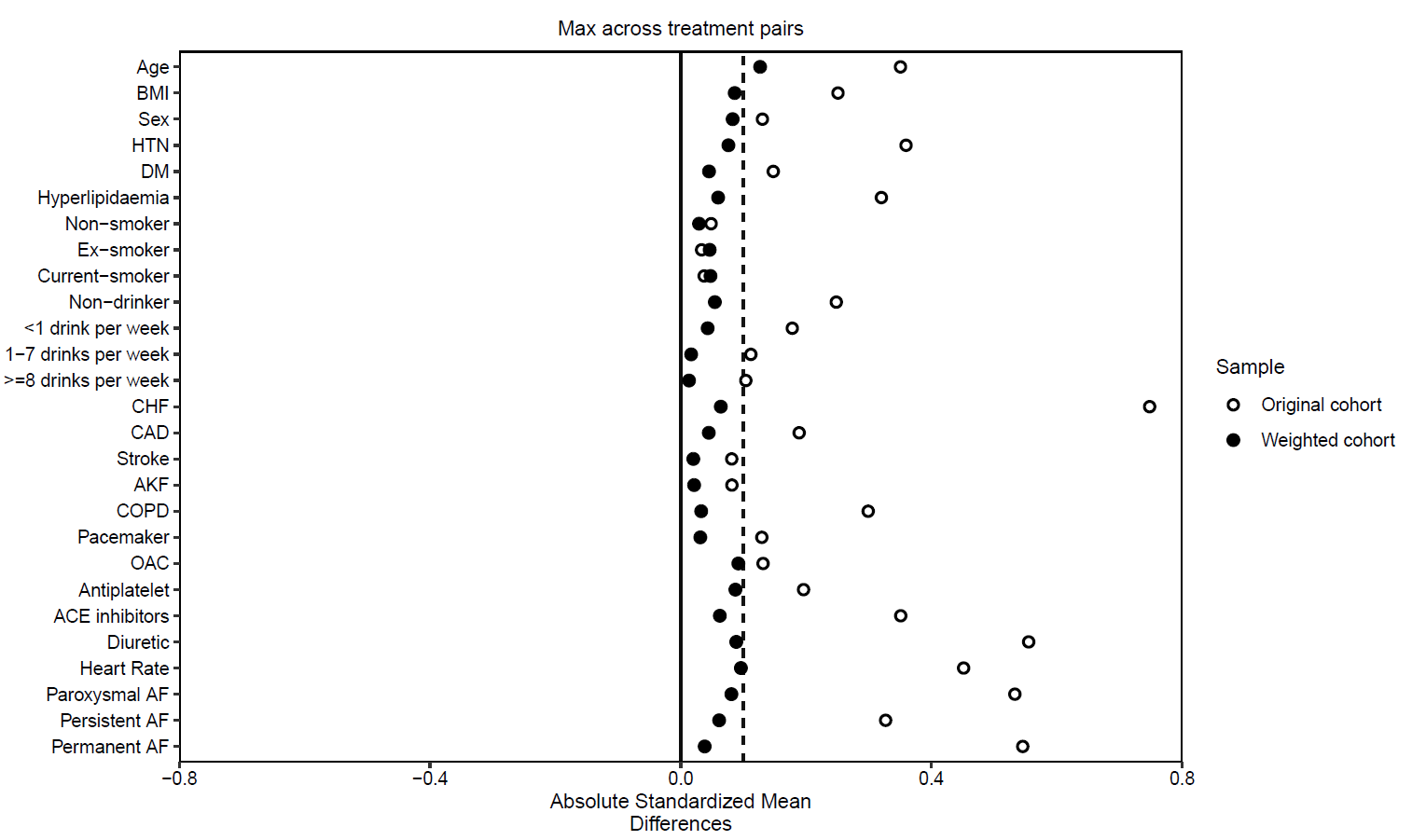


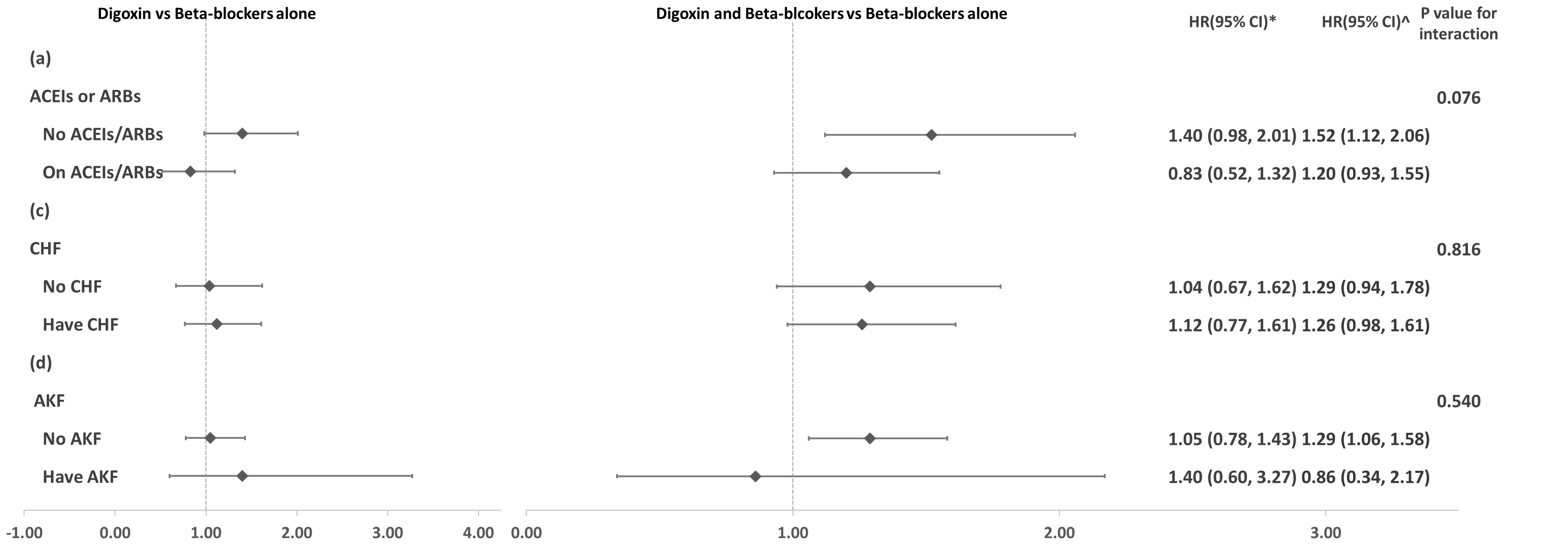


**Supplementary figure 4. Post-hoc interaction analysis for revealing the risks associated with digoxin and/or beta-blockers in different sub-groups of patients.***Hazard ratio and 95% confidence interval for comparing patients with digoxin to patients with beta-blockers alone. ^Hazard ratio and 95% confidence interval for comparing patients with digoxin and beta-blockers to patients with beta-blockers alone.

ACEIs, angiotensin-converting enzyme inhibitors; ARBs, angiotensin receptor blockers; CHF, chronic heart failure; AKF; abnormal kidney function
